# Fullerenol inhibits tendinopathy by alleviating inflammation

**DOI:** 10.3389/fbioe.2023.1171360

**Published:** 2023-03-30

**Authors:** Xin Jiao, Zengguang Wang, Yiming Li, Tianchang Wang, Chen Xu, Xianhao Zhou, Yaokai Gan

**Affiliations:** ^1^ Department of Orthopaedic Surgery, Shanghai Ninth People’s Hospital, Shanghai Jiao Tong University School of Medicine, Shanghai, China; ^2^ Shanghai Key Laboratory of Orthopaedic Implants, Shanghai Ninth People’s Hospital, Shanghai Jiao Tong University School of Medicine, Shanghai, China

**Keywords:** fullerenol, inflammation, ROS, MAPK, tendinopathy

## Abstract

Tendinopathy is a common disease in orthopaedics, seriously affecting tendon functions. However, the effects of non-surgical treatment on tendinopathy are not satisfactory and surgical treatments possibly impair the function of tendons. Biomaterial fullerenol has been proved to show good anti-inflammatory effects on various inflammatory diseases. For *in vitro* experiments, primary rat tendon cells (TCs) were treated by interleukin-1 beta (IL-1β) combined with aqueous fullerenol (5, 1, 0.3 μg/mL). Then inflammatory factors, tendon-related markers, migration and signaling pathways were detected. For *in vivo* experiments, rat tendinopathy model was constructed by local injection of collagenase into Achilles tendons of rats and fullerenol (0.5, 1 mg/mL) was locally injected 7 days after collagenase injection. Inflammatory factors and tendon-related markers were also investigated. Fullerenol with good water-solubility showed excellent biocompatibility with TCs. Fullerenol could increase expression of tendon-related factors (Collagen I and tenascin C) and decrease expression of inflammatory factors (matrix metalloproteinases-3, MMP-3, and MMP-13) and reactive oxygen species (ROS) level. Simultaneously, fullerenol slowed the migration of TCs and inhibited activation of Mitogen-activated protein kinase (MAPK) signaling pathway. Fullerenol also attenuated tendinopathy *in vivo*, including reduction of fiber disorders, decrease of inflammatory factors and increase of tendon markers. In summary, fullerenol is a promising biomaterial that can be used to treat tendinopathy.

## 1 Introduction

Tendinopathy is chronic disorders of tendons usually caused by overuse, with an incidence of 0.2%–0.3% of adult patients (van der Vlist et al., 2020). Among them, athletes are the riskiest ones to suffer from tendinopathy with a morbidity of approximate 52% (Kujala et al., 2005) (Lagas et al., 2020). Tendinopathy causes pain, diffuse or localized swelling, loss of tissue integrity and impaired performance (Millar et al., 2021). The pathological mechanisms of tendinopathy are multiple, including apoptosis disorder, mechanical overload, imbalance of matrix metalloproteinases (MMPs) and tissue inhibitors of metalloproteinases (TIMPs), genetic factors, inflammation (Yuan et al., 2002) (Arnoczky et al., 2004) (Mokone et al., 2006). Current managements of tendinopathy consist of drug treatments, physical therapy, and surgery. However, curative effects of drug treatments and physical therapies are short-term. Surgeries possibly lead to secondary injury and tendon function postoperatively cannot recover to preoperative level. Thus, it is necessary to develop a new treatment method with minor injury.

Pathology of tendinopathy still remains controversial. Inflammation plays a key role in the appearance of tendinopathy, especially in the early phase (Legerlotz et al., 2012). From the perspective of risk factors, injuries, repetitive mechanical overloading and hypoxia all elevate inflammatory cytokines, such as tumor necrosis factor alpha (TNF-α), interleukin-1 beta (IL-1β), prostaglandin E2 (PGE2) (D’Addona et al., 2017). Moreover, hypoxic damage or increased oxygen demand of tendon cells caused by mechanical stresses also tends to raise oxygen free radicals like reactive oxygen species (ROS), leading to secondary damage of tendon tissues. Simultaneously, Dakin et al. found that both tendinopathic and ruptured Achilles tendons of human expressed many CD14^+^ and CD68^+^ cells and showed a complex inflammation signature, involving interferon, nuclear factor-kappa B (NF-κB) and signal transducer and activator of transcription 6 (STAT-6) activation pathways, which also proved that inflammation was a vital pathological process of tendinopathy (Dakin et al., 2018). Therefore, inhibiting inflammation is possibly an effective method to attenuate tendinopathy.

Fullerenol is a fullerene derivative with good water solubility, which expands its use in biological and medical fields. Structurally, there are numerous carbon-carbon double bonds in fullerenol, contributing to its antioxidative activity of scavenging reactive oxygen species (ROS) (Markelić et al., 2022). A lot of studies have reported good protective effects of fullerenol on cells under oxidative stress and DNA damage. It was found that fullerenol showed excellent curative or preventive effects on bleomycin-induced pulmonary fibrosis (Zhou et al., 2018), intervertebral disk degeneration (Yang et al., 2014a), myocardial ischemia-reperfusion injury (Ding and Li, 2020), osteoarthritis (Pei et al., 2019). In the meantime, fullerenol has the ability to rescue HaCaT human skin keratinocytes and corneal epithelial cells from ultraviolet B (wavelength between 280 and 320 nm) (Saitoh et al., 2011) (Chen et al., 2022). Apart from these, fullerenol presents promising results of osteogenic differentiation induction to repair bone defect. Despite of the favorable therapeutic benefits of fullerenol on multiple diseases, there is no study on the effects of fullerenol on tendinopathy.

Since inflammation was an important feature of tendinopathy, and fullerenol showed brilliant anti-inflammatory and antioxidant effects in various diseases, we hypothesized that fullerenol could mitigate tendinopathy by inhibiting inflammation. Therefore, this study aims to explore the effects of fullerenol on tendinopathy and investigate the potential mechanisms, in order to provide a new treatment method of tendinopathy.

## 2 Materials and methods

### 2.1 Characterization of fullerenol

Fullerenol powder was purchased from Chengdu Zhongke Times Nano Energy Tech Co., Ltd. The fullerenol powder was tested by Transmission Electron Microscope (TEM, TF20) for size and morphology and by Fourier transform infrared spectrometer (FTIR, Thermo Scientific Nicolet iS20, United States). At room temperature, fullerenol was suspended in distilled water to make aqueous fullerenol with the concentration of 50 mg/mL. Then, size distribution and zeta potential were investigated by Nano Sizer and Zeta potential Tester (Omni, United States). 50 mg/mL aqueous fullerenol was stored at room temperature shielded from light for further use. For cell treatment, aqueous fullerenol was diluted with Dulbecco’s modified Eagle’s medium (DMEM, Gibco, United States) to the concentration of 10, 5, 3, 1, 0.5, 0.3, 0.1 μg/mL and was sterilized with 0.22 μm filter membranes (Millipore, United States).

### 2.2 Tendon cells isolation and culture

Tendon cells (TCs) were isolated from the Achilles tendons of rats, as described previously (Jiao et al., 2022b). In brief, Achilles tendons of one-week-old rats were cut after disinfection. And tendons were immersed in 0.06% collagenase type I (Worthington, United States) solution at 37°C overnight. Then, the solution was centrifuged and the supernatant was discarded. The sediment was suspended and incubated in DMEM supplemented with 10% fetal bovine serum (FBS, Gibco, United States ) and 1% antibiotics (penicillin and streptomycin, Gibco, United States). Cells were subcultured when they reached 80%–90% confluence.

### 2.3 Cell Count Kit-8 assay

Cell Count Kit-8 (CCK-8) assay was performed using the kit (Dojindo, CK04-05, Japan) according to the instruction. TCs were seeded into 96-well plates with a density of 3 × 10^3^ per well. Then, TCs were incubated using DMEM containing fullerenol with different concentrations (10, 5, 3, 1, 0.5, 0.3, 0.1 μg/mL). At 1 and 3 days, TCs were cultured in DMEM medium with 10% CCK-8 reagent at 37°C for 2 h. The absorbance of the supernatant at 450 nm was measured using a microplate reader (Infinite M200 Pro, Tecan, Switzerland).

### 2.4 Live/dead cell staining

Live/dead cell staining was performed using the kit (KeyGEN, Nanjing, China) according to the manufacturer’s instruction. The samples were observed using a confocal microscope (Leica, Germany). Live (green) cells stained by Calcein AM were detected with excitation at 488 nm, and dead (red) cells stained by PI were observed with excitation at 555 nm.

### 2.5 RNA extraction and qRT-PCR

For inflammation induction and fullerenol treatment, TCs were treated by 50 ng/mL IL-1β combined with aqueous fullerenol. Then, total RNA was extracted using TRIzol reagent (Thermo Scientific, United States). A NanoDrop 1,000 spectrophotometer (Thermo Scientific, United States) was used to evaluate RNA purity and quantification. 1,000 ng of the extracted RNA was reverse transcribed to cDNA using PrimeScript Master Mix (Takara, RR036A, Japan). The qRT-PCR reaction was performed with 2× SYBR Green qPCR Master Mix (Low ROX) (Bimake, B21703, China) and Applied Biosystems 7,500 Real-Time PCR System (Applied Biosystems, Foster City, CA, United States). The relative mRNA levels were calculated with 2^−ΔΔCT^ method. Glyceraldehyde-3-phosphate dehydrogenase (GAPDH) was used as internal control. The primers used in this study are listed in Table 1.

**TABLE 1:** Sequences of primers for qRT-PCR.

Primer	Forward primer (5′ to 3′)	Reverse primer (5′ to 3′)
GAPDH	GGC​AAG​TTC​AAC​GGC​ACA​GT	GCC​AGT​AGA​CTC​CAC​GAC​AT
COL1A1	TGA​CTG​GAA​GAG​CGG​AGA​GTA	GGG​GTT​TGG​GCT​GAT​GTA​CC
TNC	TGC​CAT​AGC​AAC​AAC​AGC​CAT	AAC​TCT​CCA​CCT​GAG​CAG​TC
MMP-3	TGC​TCA​TGA​ACT​TGG​CCA​CT	GTG​GGA​GGT​CCA​TAG​AGG​GAT
MMP-13	GGG​AAC​CAC​GTG​TGG​AGT​TAT	GAC​AGC​ATC​TAC​TTT​GTC​GCC

**Abbreviations:** GAPDH, glyceraldehyde-3-phosphate dehydrogenase; COL1A1, collagen 1A1; TNC, tenascin C; MMP-3, matrix metalloproteinases-3; MMP-13, matrix metalloproteinases-13.

### 2.6 Scratch assay

For scratch assay, TCs were seeded into 6-well plates and cultured to reach 80% confluence. Then, a straight scratch was scraped with a 200-μL pipette tip. And TCs were incubated with IL-1β and aqueous fullerenol. Cell migration was determined by measuring the distance at 0, 12 and 24 h.

### 2.7 Transwell assay

For the Transwell assay, Transwell chambers (BD Science, United States of America) were used. In 24-well plate, TCs (5 × 10^4^) in 150 μL of serum-free basal medium were seeded into the upper chamber, and 650 μL of DMEM supplemented with 10% FBS, IL-1β and aqueous fullerenol was added into the lower chamber. The Transwell system was placed in a 5% CO_2_ incubator at 37 C for 24 h. Then, the cells were fixed and stained with crystal violet solution.

### 2.8 Protein extraction and Western blotting

For inflammation induction and fullerenol treatment, TCs were treated by 50 ng/mL IL-1β combined with aqueous fullerenol. For protein extraction, TCs were lysed using RIPA lysis buffer (Beyotime, China) supplemented with 1% protease and phosphatase inhibitor cocktail (100X) (Thermo Scientific, United States). Then, the mixture was centrifugated at a speed of 14,000 RCF for 15 min. The supernatant was separated and mixed with SDS-PAGE sample loading buffer (Beyotime, China) and boiled at 99°C for 5 min. Protein samples were electrophoresed on SDS gels and transferred onto polyvinylidene fluoride membranes (Millipore, United States). The membrane was then blocked in Tris-buffered saline Tween 20 (Solarbio, China) containing 5% non-fat milk (Sangon Biotech, China) or 5% bovine serum albumin (MPbio, United States) for 1 h at room temperature. After that, the membrane was incubated with primary antibodies at 4°C overnight and secondary antibodies for 1 h at room temperature. Protein immunoreactivity was detected with LI-COR Odyssey Fluorescence Imaging System (LI-COR Biosciences, United States), and ImageJ was used to measure the protein expression. The anti-bodies used were as follows: p44/42 MAPK (Erk1/2) (Cell Signaling Technology, United States), Phospho-p44/42 MAPK (Erk1/2) (Cell Signaling Technology, United States), SAPK/JNK (Cell Signaling Technology, United States), Phospho-SAPK/JNK (Cell Signaling Technology, United States), p38 MAPK (Cell Signaling Technology, United States), Phospho-p38 MAPK (Cell Signaling Technology, United States), Anti-rabbit IgG (H + L) (800 4X PEG Conjugate) (Cell Signaling Technology, United States), Anti-mouse IgG (H + L) (800 4X PEG Conjugate) (Cell Signaling Technology, United States).

### 2.9 Animal experiments

All animal experiments were approved by the Ethics Committee of Shanghai Ninth People’s Hospital, Shanghai Jiaotong University School of Medicine (Approval number: SH9H-2021-A895-1). To establish tendinopathy models, Sprague-Dawley (SD) rats (male, 8 weeks old), purchased from Shanghai JieSiJie Laboratory Animals Co., LTD., were anesthetized. Then, 50 mg/mL collagenase type I (Worthington, United States) solution was injected into the Achilles tendons to trigger inflammation. At 7 days after injection, 50 μL aqueous fullerenol with the concentration of 0.5 mg/mL and 1 mg/mL were injected. At the 21st day after collagenase injection, tendons were collected and used for histological observation.

### 2.10 Histological observation

The histological observation methods were similar to those previously reported (Jiao et al., 2022a). Briefly, after fixation, embedding and section, Hematoxylin-eosin (HE) and Masson tri-chrome staining were performed. The method of evaluating fiber alignment was described in the previous studies (Adeoye et al., 2022) (Ozlu et al., 2019) (Erisken et al., 2013). For immunohistochemical staining, we incubated sections overnight with different antibodies (COL I, COX-2, IL-6; Servicebio; China). On the next day, the sections were incubated with the secondary antibody (HRP-anti-rabbit IgG, Servicebio, China). After that, they were observed and captured.

### 2.11 Statistical analysis

All results are shown as the mean ± standard deviation. Student’s t-test was used for comparisons between two groups, and one-way analysis of variance followed by Tukey’s *post hoc* analysis was used for comparisons between three or more groups. Statistical significance was set at *p* < 0.05.

## 3 Results

### 3.1 Characterization of fullerenol

**FIGURE 1:**
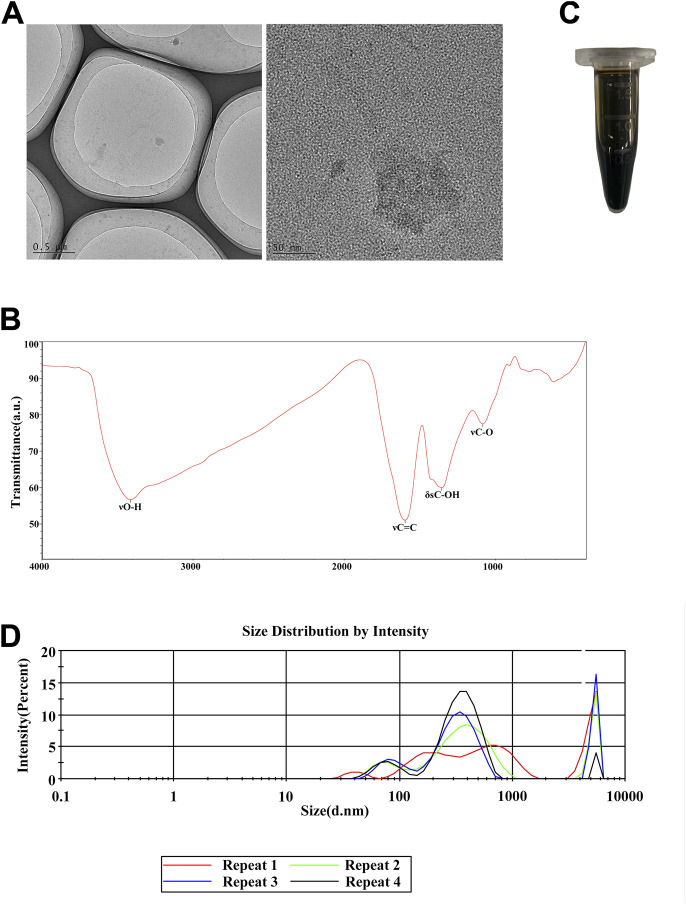
Characterization of fullerenol. **(A)** Transmission electron microscope (TEM) of fullerenol powder. Scale bar = 0.5 μm (left), 50 nm (right) **(B)** FTIR of fullerenol powder. **(C)** Image of aqueous fullerenol (50 mg/mL). **(D)** Hydrodynamic size of fullerenol in aqueous solution.

To detect the characterization of fullerenol, we investigated the size and morphology of fullerenol powder by TEM. Shown in Figure 1A, the diameter of fullerenol powder was over 1 μm. Furthermore, we detected characteristic absorption peaks of fullerenol powder by FTIR spectra. In Figure 1B, four characteristic absorption peaks existed. In detail, broad O–H stretching vibration (νO-H) presented at 3,412.80 cm^-1^, C=C stretching vibration (νC = C) was shown at 1,596.71 cm^-1^, O–H in-plane deformation vibration (δsC-OH) existed at 1,354.07 cm^-1^, and C–O stretching vibration (νC-O) was at 1,082.82 cm^-1^. Then, we dissolved fullerenol by water into the concentration of 50 mg/mL, which became brown to black liquid (Figure 1C). Although the diameter of fullerenol powder was over 1μm, according to size distribution, particles in aqueous fullerenol were mostly from 100 to 1000 nm (Figure 1D). Notably, there was another peak from 3,000 to 6000 nm, which was probably caused by agglomeration due to high concentration. At the same time, the surface zeta potential was −15.44 ± 1.93 mV (Table 2).

**TABLE 2:** Zeta potential of aqueous fullerenol.

Repeat	Zeta potential (mV)	Mobility (μ/s)/(V/cm)	Conductance (μS)	Count rate (kcps)
1	−16.61	−1.30	25	586
2	−13.21	−1.03	26	601
3	−16.51	−1.29	26	601

### 3.2 Fullerenol shows low cytotoxicity on rat TCs

**FIGURE 2:**
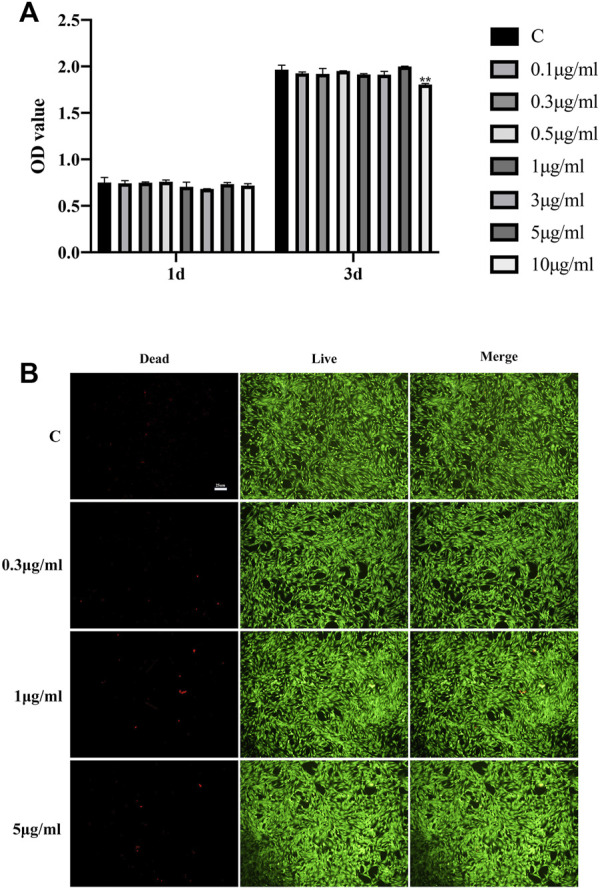
Biocompatibility of fullerenol with different concentrations. **(A)** Optical density (OD) value of TCs treated with different-concentration (0, 0.1, 0.3, 0.5, 1, 3, 5, 10 μg/mL) fullerenol at 1 and 3 days tested by CCK-8. **(B)** Live/dead cell staining of TCs treated with 0, 0.3, 1, 5 μg/mL fullerenol at 3 days. Scale bar = 25 μm. (Data are presented as the mean ± standard deviation. *p < 0.05, **p < 0.01).

To detect the cytotoxicity of fullerenol, we performed CCK-8 assay. Shown in Figure 2A, at 1 day after fullerenol (10, 5, 3, 1, 0.5, 0.3, 0.1 μg/mL) treatment, no significant difference existed between TCs treated with fullerenol and without fullerenol, indicating no cytotoxicity at 1 day. However, at 3 days, optical density (OD) value in the 10 μg/mL group was obviously lower than control group, suggesting that 10 μg/mL fullerenol influenced cell viability of TCs at 3 days. Based on this, we chose three concentrations (5, 1, 0.3 μg/mL) to conduct further experiments. Furthermore, we verified the cytotoxicity of fullerenol with the three concentrations at 3 days *via* live/dead cell staining (Figure 2B). It was found that almost no dead TCs existed at the three concentrations. All the results showed that low-concentration fullerenol had good cytocompatibility with TCs.

### 3.3 Fullerenol inhibits inflammation of TCs caused by IL-1β and rescues the impairments of TCs

**FIGURE 3:**
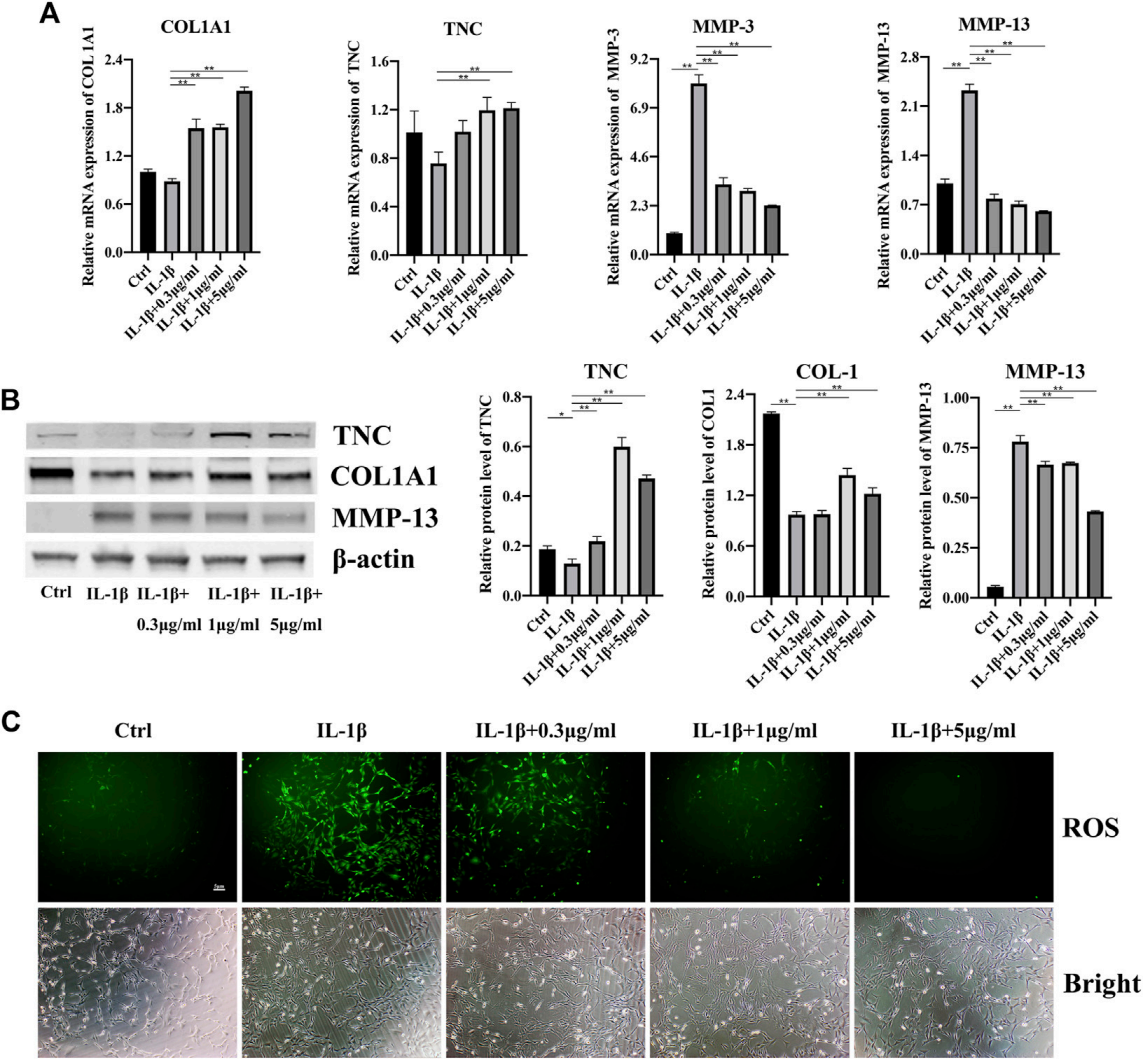
Effects of fullerenol on inflammation of TCs induced by IL-1β. **(A)** mRNA level of COL1A1, TNC, MMP-3 and MMP-13 of TCs after fullerenol and IL-1β treatment tested by qRT-PCR. **(B)** Protein level of TNC, COL1A1 and MMP-13 of TCs after fullerenol and IL-1β treatment tested by Western Blot (Left) and quantitative results (Right). **(C)** ROS level of TCs after fullerenol and IL-1β treatment tested by ROS assay kit.

Next, we investigated the effects of fullerenol on the inflammation of TCs and the expression of tendon-related markers. Collagen 1A1 (COL1A1) is the most important component of tendon tissues and expresses lower in tendinopathy (Cho et al., 2021; López De Padilla et al., 2021). Tenascin C (TNC) is a glycoprotein abundantly expressed in tendons subjected to high tensile and compressive stress (September et al., 2007). TNC has been proved in the regulation of cell-matrix interaction (September et al., 2007). Shown in Figure 3A, after adding IL-1β, expression of COL1A1 and TNC decreased, although there was no significant difference. Fullerenol enhanced the RNA level of COL1A1 and TNC remarkably, especially 5 μg/mL. Contrary to COL1A1 and TNC, IL-1β augmented matrix metalloproteinases-3 (MMP-3) and matrix metalloproteinases-13 (MMP-13) expression, which were closely related to inflammation. As an anti-inflammatory material, fullerenol lowered MMP-3 and MMP-13, suggesting that fullerenol alleviated inflammation. Consistent with RNA, the tendency of TNC, COL I and MMP-13 were increased by IL-1β and decreased by fullerenol (Figure 3B). In view of anti-oxidant effects of fullerenol, we also verified the anti-oxidant effect of fullerenol in tendinopathy. In Figure 3C, IL-1β induced ROS upregulation, showing that IL-1β exacerbated oxidant stress in TCs. However, after fullerenol treatment, ROS level of TCs diminished in a concentration-dependent manner and nearly disappeared in the concentration of 5 μg/mL. All the above results implied that fullerenol could attenuate inflammation and ROS level in TCs induced by IL-1β.

### 3.4 Fullerenol inhibits migration of TCs

**FIGURE 4:**
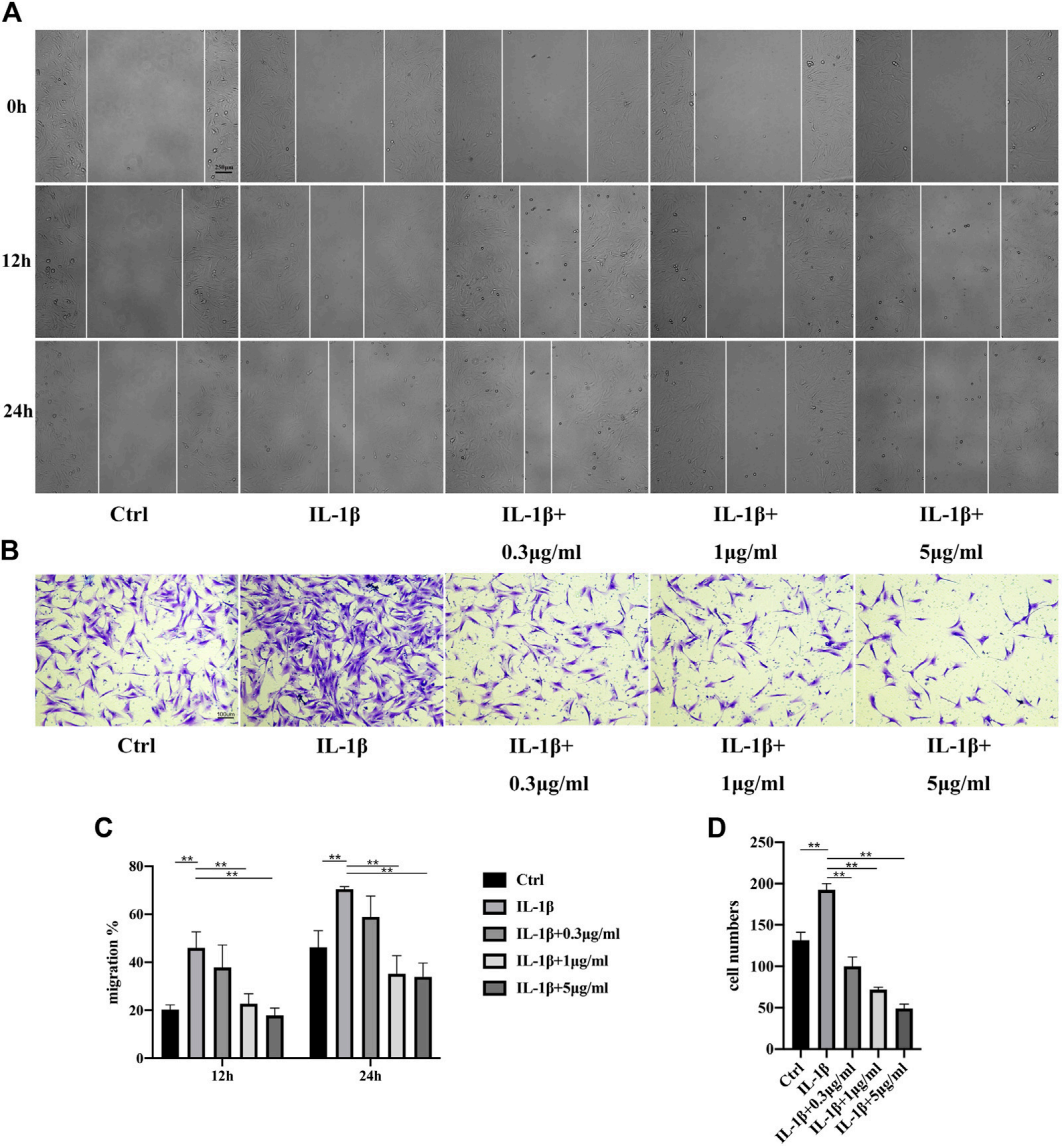
Migration of TCs after IL-1β (50 ng/mL) and fullerenol treatment. **(A)** Migration of TCs in control (Ctrl), IL-1β, IL-1β+0.3 μg/mL, IL-1β+1 μg/mL, IL-1β+5 μg/mL groups tested by scratch assay. Scale bar = 250 μm. **(B)** Migration of TCs in Ctrl, IL-1β, IL-1β+0.3 μg/mL, IL-1β+1 μg/mL, IL-1β+5 μg/mL groups tested by transwell assay. Scale bar = 100 μm. **(C)** Quantitative results of scratch assay. **(D)** Quantitative results of transwell assay. (Data are presented as the mean ± standard deviation. *p < 0.05, **p < 0.01).

It was reported that migration of TCs increased in an inflammatory environment (Jiao et al., 2022b) (Wang et al., 2019). Next, we investigated the influences of fullerenol on TCs migration through scratch assay and transwell assay. Shown in Figures 4A,C, TCs in all the five groups migrated gradually at 12 and 24 h. The addition of IL-1β accelerated migration of TCs to around 50% at 12 h and approximate 70% at 24 h. However, fullerenol was able to inhibit the migration of TCs effectively. Notably, the inhibitory effects were concentration dependent. Extremely low concentration like 0.3 μg/mL did not depress the migration of TCs, while 1 μg/mL and 5 μg/mL could suppress the migration. Similarly, in transwell assay, the number of TCs in IL-1β group increased obviously compared with control group (Ctrl). But the number declined after fullerenol treatment in a concentration-dependent manner (Figures 4B,D). The above results implied that fullerenol could effectively inhibit migration of TCs.

### 3.5 Fullerenol inhibits tendinopathy *via* MAPK pathway

**FIGURE 5:**
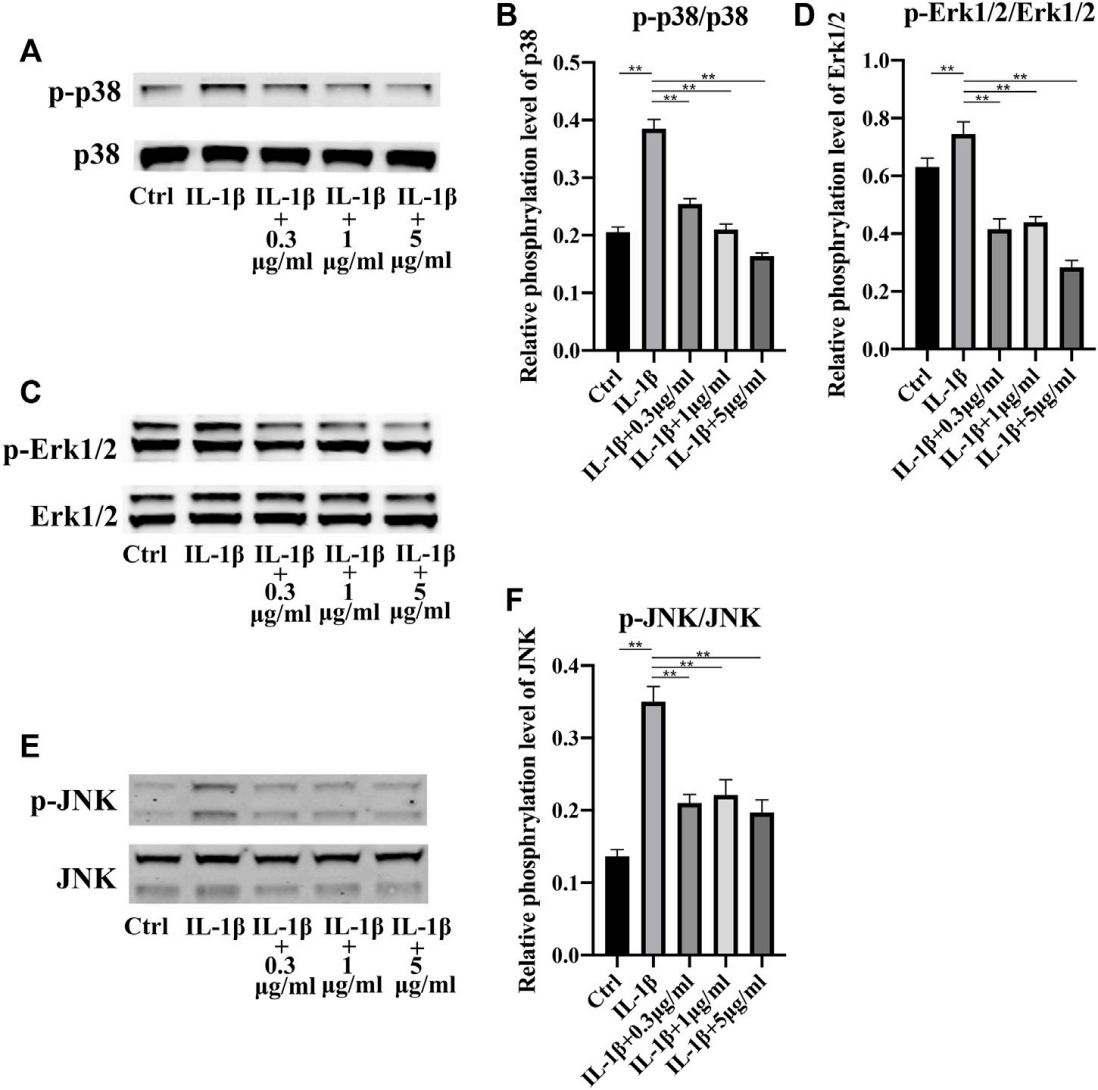
Expression of MAPK signaling pathway after IL-1β (50 ng/mL) and fullerenol (0.3, 1, 5 μg/mL) treatment. **(A)** The phosphorylation levels of p38 in TCs of Ctrl, IL-1β, IL-1β +0.3 μg/mL, IL-1β+1 μg/mL, IL-1β+5 μg/mL groups were examined by Western Blotting. **(B)** Quantitative results of phosphorylation levels of p38. **(C)** The phosphorylation levels of Erk1/2 in TCs of Ctrl, IL-1β, IL-1β +0.3 μg/mL, IL-1β+1 μg/mL, IL-1β+5 μg/mL groups were examined by Western Blotting. **(D)** Quantitative results of phosphorylation levels of Erk1/2. **(E)** The phosphorylation levels of JNK in TCs of Ctrl, IL-1β, IL-1β +0.3 μg/mL, IL-1β+1 μg/mL, IL-1β+5 μg/mL groups were examined by Western Blotting. **(F)** Quantitative results of phosphorylation levels of JNK. (Data are presented as the mean ± standard deviation. *p < 0.05, **p < 0.01)

Mitogen-activated protein kinase (MAPK) signaling pathway was reported to play a key role in inflammation (Jiao et al., 2022b) (Zhu et al., 2021). So, we explored the activation of MAPK signaling pathway. P38 MAPK pathway was strongly activated in stress, immune response and regulation of cell survival and differentiation (Cuadrado and Nebreda, 2010). Apparently, in our study, inflammation induced by IL-1β increased the phosphorylation level of p38. Nevertheless, addition of fullerenol availably hindered the activation of p38 (Figures 5A,B). Interestingly, phosphorylation level of p38 decreased in a concentration-dependent manner. Identically, Erk1/2 and JNK was activated by IL-1β and the activation was inhibited by fullerenol (Figures 5C–F). The results of Western blot suggested that fullerenol could restrain the activation of MAPK pathway induced by IL-1β.

### 3.6 Fullerenol inhibits tendinopathy *in vivo*

**FIGURE 6:**
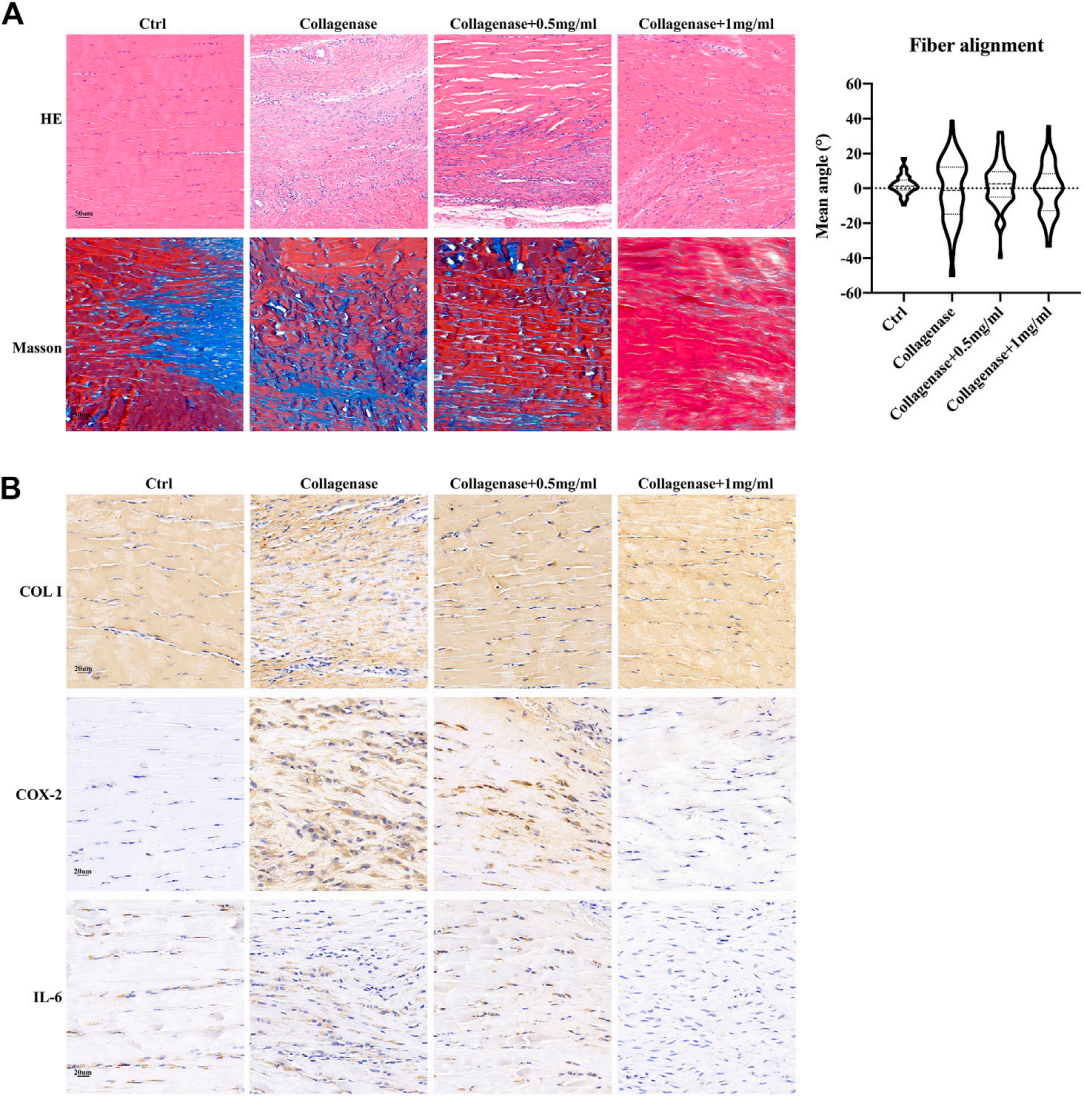
Inhibition of fullerenol (0.5, 1 mg/mL) on tendinopathy *in vivo*. **(A)** HE and Masson staining of tendons in Ctrl, Collagenase, Collagenase+0.5 mg/mL, Collagenase+1 mg/mL groups (Left). Statistical results of fiber alignment (Right). Scale bar = 50 μm. **(B)** Immunohistochemical staining (COL I, COX-2, IL-6) of tendons in Ctrl, Collagenase, Collagenase+0.5 mg/mL, Collagenase+1 mg/mL groups. Scale bar = 20 μm.

Next, we furtherly tested anti-inflammatory effects of fullerenol on tendinopathy *in vivo*. Shown in Figure 6A, after collagenase I injection, tendinous fibers were fractured and arranged disorderly compared with Ctrl group in HE and Masson staining. But fullerenol alleviated impairment of tendinous fibers. Meanwhile, we detected expression of Collagen I (COL I), Cyclooxygenase 2 (COX-2) and IL-6 by immunohistochemical staining (Figure 6B). In Collagenase group, COL I decreased and inflammatory factors (COX-2 and IL-6) increased in comparison with Ctrl group, showing that collagenase induced inflammation of tendon tissues. Fullerenol could alleviate severity of inflammation and promote expression of COL I. All these data hinted that fullerenol reduced inflammation in tendinopathy *in vivo*.

## 4 Discussion

Tendinopathy is a common overload injury, with an incidence of two to three per 1,000 patients in general medicine practice (van der Vlist et al., 2021). It is challenging to manage tendinopathy. Current treatments have more or less limitations. For example, conservative treatments like eccentric exercises and shockwave therapy are not suitable for all kinds of tendinopathy (Figueroa et al., 2016). Pharmacological management, especially injection, is another important way to treat tendinopathy. Unfortunately, no standard procedure of treat tendinopathy pharmacologically is established because there is a lack of comparative studies on effects of various drug injections (Aicale et al., 2020). Furthermore, surgical treatment impaired the function of tendons and there is a need of high-quality evidence on the effects of surgeries on different tendinopathy, such as chronic patellar tendinopathy (Khan and Smart, 2016). Therefore, it is necessary to develop a new method of tendinopathy management with low side effects.

Fullerenol is a hydroxylated derivative of fullerene. Identical to previous studies (Wang et al., 2016) (Zha et al., 2022), the diameter of our aqueous fullerenol ranged from 100 to 1,000 nm. Notably, fullerenol was also manufactured to be nanomaterial with diameter lower than 100 nm (Chen et al., 2022). In terms of biological function, fullerenol show good biocompatibility and low side effects in numerous studies (Yang et al., 2021) (Yang et al., 2014b) (Zhu et al., 2007). However, it was also reported that fullerenol was cytotoxic toward human retinal pigment epithelial (hRPE) cells at concentrations of 10–50 μM, and increased phototoxicity on hRPE cells in particular (Wielgus et al., 2010). So, in spite of good biocompatibility, fullerenol is not absolutely safe to all the normal tissue cells. In our study, we detected the cytotoxicity of fullerenol toward TCs. It was found that after short-term (1 day) treatment, fullerenol showed no cytotoxicity at concentrations of 0.1–10 μg/mL. Nonetheless, with extension of treatment time to 3 days, fullerenol at high concentration (10 μg/mL) became deleterious for cell viability of TCs. Overall, fullerenol, consisting of carbon, hydrogen and oxygen elements, exhibited good biocompatibility to TCs. But the extremely high concentration also caused impairment on TCs. All the results of cell viability are almost identical to previous studies.

Inflammation is one of the most features of tendinopathy and suppressing inflammation has been a vital treatment for tendinopathy. Mounting studies have employed anti-inflammatory drugs and biomaterials to treat tendinopathy. Chen et al. reported that ibuprofen-loaded hyaluronic acid nanofibrous membranes could reduce inflammation to prevent postoperative tendon adhesion (Chen et al., 2019). In the study of Choi et al., they synthesized lactoferrin-immobilized, heparin-anchored, poly (lactic-co-glycolic acid) nano-particles (LF/Hep-PLGA NPs) and also found that LF/Hep-PLGA NPs enhanced tendon restoration *via* inhibiting inflammation (Choi et al., 2020). In terms of drugs, plenty of drugs like aspirin were found to be conducive to tendinopathy treatment through many signaling pathways, such as JNK/STAT3 pathway (Wang et al., 2019). In our study, we investigated whether fullerenol helped mitigate tendinopathy. From RNA to protein level, under inflammatory environment, fullerenol increased expression of COL I and TNC and decreased expression of MMP-3 and MMP-13. It might imply that fullerenol played a key role in both reducing inflammation and protecting tendon tissues. Furthermore, production of ROS is another factor of damage to tendon. The results of our study showed that fullerenol also had the ability to remove ROS, which was possibly due to many carbon-carbon double bonds in fullerenol.

Stimulation of inflammation tends to change the behaviors of localized cells in different tissues. Cell migration is one of the most important behaviors affected by inflammation. In previous studies, it was found that the migration of human bronchial epithelial cells increased after being treated with TNF-α, which was also a way to induce inflammation (Ren et al., 2021). Similarly, migration of fibroblasts is also influenced by inflammation. Fibroblast-like synoviocytes (MH7A cell) in rheumatoid arthritis migrated faster than synoviocytes in control group (Cai et al., 2021) (Cai et al., 2022). Zhang et al. also reported that macrophage migration inhibitory factor, a proinflammatory cytokine, promoted migration of joint capsule fibroblasts (Zhang et al., 2021). Here, in our study, we investigated the influence of fullerenol on TCs migration under the inflammatory environment by scratch assay and transwell assay. Obviously, IL-1β treatment induced inflammation of TCs successfully and made migration of TCs faster, which was consistent with our previous study (Jiao et al., 2022b). In view of good anti-inflammatory and anti-oxidant effects of fullerenol on TCs from mRNA level to protein level, it could inhibit migration of TCs under inflammatory environment, unsurprisingly. After tendons are injured, migration of TCs may lead to formation of a fibrotic scar, causing loss of mechanical strength of the original tendon (Wang et al., 2019) (Nichols et al., 2019). Based on our results, fullerenol slowed the migration of TCs which was likely to reduce the impairment of tendon tissues.

A series of signaling pathways is of great importance in tendinopathy inhibition. Various inflammation-related pathways such as NF-κB, c-Jun N-terminal kinase (JNK)/STAT-3 signaling pathways (Wang et al., 2019) (Vinhas et al., 2020). Besides, MAPK signaling pathways play a part in tendinopathy (Wu et al., 2022) (Moqbel et al., 2020). MAPK cascade consists of three protein kinases, including a MAPK and two upstream components, MAPK kinase (MAPKK) and MAPKK kinase (MAPKKK). So far, three MAPK pathways are found in mammalian cells, namely, the extracellular signal-regulated kinases (ERKs) pathway, the c-Jun amino terminal kinase (JNK) pathway and the p38 MAPK pathway (Kumar et al., 2003). Considering the importance of MAPK signaling pathway in inflammation, we explored the activation of MAPK pathway after fullerenol treatment. Excitingly, fullerenol curbed the phosphorylation level of all p38, ERK, and JNK, suggesting fullerenol could effectively inhibit tendinopathy *via* MAPK pathway.

We furtherly detected the effects of fullerenol on tendinopathy *in vivo*. Collagenase injection has been a common method of constructing tendinopathy model (Liu et al., 2021) (Wu et al., 2019). Collagenase injection caused disorders and swelling of fibers in tendons. At the same time, collagenase leads to increase of inflammatory factors and decrease of Collagen I. Since fullerenol had good water-solubility, we decided to inject fullerenol locally into Achilles tendons, which had better effects for tendinopathy compared with intraperitoneal injection. In our study, identical to results of cell experiments, aqueous fullerenol alleviated disorders of tendon fibers and decreased expression of inflammatory factors like COX-2 and IL-6. The animal experiments showed fullerenol was an excellent and convenient therapeutic approach to tendinopathy.

Since tendinopathy is a localized inflammatory disease, traditional drug administration is the most common treatment. Here, we firstly injected aqueous fullerenol to treat tendinopathy. In view of good biocompatibility and anti-inflammatory effects, fullerenol shows good prospects in treating localized inflammatory diseases like tendinopathy in the future.

## 5 Conclusion

In conclusion, fullerenol is a promising biomaterial which has brilliant biocompatibility and anti-inflammatory effects and can be used to treat tendinopathy. Utilization of fullerenol helps reduce the side effects caused by drug administration and lower the economic burden.

## Data Availability

The raw data supporting the conclusion of this article will be made available by the authors, without undue reservation.
